# Effect and mechanism of resveratrol against polycystic ovary syndrome: a review

**DOI:** 10.3389/fendo.2025.1529231

**Published:** 2025-07-07

**Authors:** Hui Chang, Baichao Shi, Yu Wang, Fengjuan Lu, Muxin Guan, Xiaoke Wu

**Affiliations:** ^1^ Department of Obstetrics and Gynecology, First Affiliated Hospital, Heilongjiang University of Chinese Medicine, Harbin, China; ^2^ Graduate School, Heilongjiang University of Chinese Medicine, Harbin, China

**Keywords:** polycystic ovary syndrome, resveratrol, mechanism, metabolism, treatment

## Abstract

Polycystic ovary syndrome (PCOS) is a prevalent reproductive endocrine disorder characterized by significant clinical heterogeneity. The etiology of PCOS remains elusive, though it is closely linked to genetic, metabolic, endocrine, and environmental determinants. Resveratrol, a natural polyphenol, has garnered considerable interest due to its diverse biological activities. While resveratrol has been shown to ameliorate disorders in glucose, lipid, and endocrine metabolism associated with PCOS, the precise underlying mechanisms remain unclear. This narrative review aims to explore the potential mechanisms through which resveratrol operates, considering the multifaceted pathological pathways of PCOS, thereby offering insights for clinical applications and drug development.

## 1 Introduction

Polycystic ovary syndrome (PCOS) is a complex, heterogeneous disease that is a primary cause of infertility among reproductive-aged women, which is characterized by clinical and/or biochemical hyperandrogenism (HA), ovulation dysfunction, and polycystic ovary morphology (PCOM) (1), alongside elevated luteinizing hormone (LH) levels and increased LH-to-follicle stimulating hormone (FSH) ratio (2). PCOS is further associated with metabolic comorbidities including obesity, insulin resistance (IR), type 2 diabetes T2D, and hepatic steatosis, collectively elevating cardiovascular risk (3, 4). The reproductive-related implications encompass menstrual irregularities, anovulatory infertility, heightened risks of pregnancy complications and endometrial cancer (5). Globally, the age-standardized prevalence and annual incidence rates of PCOS in 2019 were 1,677.8 and 59.8 per 100,000, respectively, reflecting a 30.4% and 29.5% increase since 1990 (6). Despite prevalence and incidence rising, the pathogenesis of PCOS remains incompletely elucidated. HA and IR play crucial roles in the progression of the disease (7). In other words, HA and IR are not merely common clinical manifestations of PCOS but also central pathogenic drivers of the disease (8). However, due to the heterogeneity of PCOS population and inherent racial/ethnic variations, the existing diagnostic criteria struggle to achieve universal applicability across all global regions (9), hindering the development of targeted therapies.

Current treatment for PCOS integrates lifestyle modifications with pharmacotherapy. Lifestyle optimization focuses on a balanced, nutrient-dense diet and regular physical exercise to prevent excessive weight gain, mitigate PCOS-related complications, and promote weight loss when necessary. Pharmacological strategies include metformin to improve IR and correct associated metabolic abnormalities, combined oral contraceptive pills (COCPs) for regulating menstrual cycles and HA, and if needed, anti-androgens for refractory HA (10). Fertility-focused therapies such as letrozole and clomiphene are employed for ovulation induction. Nevertheless, there medicine predominantly address symptom management and assisted fertility rather than curative outcomes (11). There are many adverse reactions including metformin-related gastrointestinal intolerance, COCPs discontinuation relapses, anti-androgen teratogenicity, and elevated miscarriage/multiple pregnancy risks with letrozole and clomiphene (12–14). Therefore, it is imperative to seek a novel drug with multi-target properties, no adverse effects on patients’ health, and potential therapeutic effects.

Resveratrol, a natural polyphenolic compound found in plants such as peanuts, grapes, knotweed, and mulberries (15), demonstrates pleiotropic biological activities. Preclinical studies highlight its capacity to improve ovarian histomorphology, regulate sex hormones and gonadotropins, modulate glucose/lipid metabolism, and exert antioxidant/anti-inflammatory effects. Clinical evidence suggests resveratrol may improve ovarian volume, increase the rate of high-quality oocytes and embryos, decrease androgen and gonadotropin concentrations, angiogenic factor levels, and alleviate endoplasmic reticulum stress in PCOS patients (16). This review aims to investigate PCOS and its related basic and clinical research, summarizing the mechanism of resveratrol in improving PCOS, with the goal of providing a reference for clinical drug administration.

## 2 Resveratrol improves abnormal glucose metabolism

IR and compensatory hyperinsulinemia are observed in 65%-95% of women with PCOS, encompassing the majority of overweight/obese individuals and over half of normal-weight women (17). Notably, IR has been identified as an independent risk factor for early miscarriage and fetal macrosomia in PCOS patients undergoing their first embryo transfer cycle, with both complication rates escalating proportionally to rising homeostasis model assessment of insulin resistance (HOMA-IR) (18). Beyond reproductive implications, IR serves as a central nexus linking cardiometabolic pathologies—including hyperglycemia, atherogenic dyslipidemia, and hypertension—thereby amplifying the risk of cardiovascular disease in adults, particularly within the PCOS population (19).

Emerging preclinical evidence demonstrates that one-month resveratrol treatment normalizes estrous cyclicity in letrozole-induced PCOS rodent models. Key outcomes include significant reductions in body weight, increases in the number of granulosa cell layers, and the presence of oocytes within follicles (20), highlighting resveratrol’s potential as a novel therapeutic agent. Translating these findings to humans, a randomized, double-blind, placebo-controlled trial involving 34 PCOS patients revealed that three-month resveratrol supplementation reduced fasting insulin (FINS) by 31.8% and improved the insulin sensitivity index (ISI) by 66.3% (21). Complementary studies in T2D populations have reported resveratrol-mediated improvements in fasting blood glucose (FBG), glycated hemoglobin (HbA1c), and HOMA-IR (22–24), underscoring its metformin-like capacity to enhance insulin sensitivity and decreases FINS concentration while uniquely regulating glucose metabolism. Intriguingly, resveratrol-metformin combination therapy has demonstrated a superior enhancement in glucose tolerance compared to metformin monotherapy (25), suggesting divergent yet complementary mechanisms of action. This synergy positions resveratrol as a promising adjunct to conventional anti-PCOS regimens.

### 2.1 Regulation of glucose metabolism-related signaling pathways

Silent information regulator 1 (SIRT1) a nicotinamide adenine dinucleotide (NAD^+^)-dependent deacetylase, catalyzes the deacetylation of histone and non-histone proteins, exerting pleiotropic effects on endocrine and metabolic regulation, immune responses, oxidative stress mitigation, inflammatory pathways, and aging (26). Dysregulation of SIRT1 has been implicated in diverse pathologies, including hyperuricemia, diabetes, hypertension, dyslipidemia, osteoporosis, and PCOS (26). Concurrently, adenine monophosphate activated protein kinase (AMPK), a cellular energy sensor activated by declining energy status (elevated AMP: ATP and ADP: ATP ratios), restores energy homeostasis by stimulating ATP- producing catabolic pathways while suppressing energy-consuming processes (27). Both SIRT1 and AMPK orchestrate critical cellular functions—including energy metabolism, cell cycle progression, and apoptosis—while modulating glucose homeostasis, glycogen synthesis, insulin secretion, and fatty acid metabolism. Emerging evidence identifies the SIRT1/AMPK axis as a pivotal molecular mechanism underlying IR in PCOS, with resveratrol’s glucoregulatory effects partially attributed to its activation of this pathway (28).

Studies have demonstrated that resveratrol may activate and upregulate NAD^+^-dependent SIRT1 which binds to the forkhead box transcription factors O1 (FOXO1), FOXO3a, and FOXO4, specifically deacetylating these proteins (29). Such deacetylation enhances the DNA-binding capacity of FOXOs to specific target gene promoters, amplifying their transcriptional activities (29). Hyperacetylation of FOXO1, conversely, exacerbates hyperglycemia and IR by potentiating FOXO1-driven gluconeogenesis. Resveratrol may counteract this by SIRT1-mediated FOXO1 suppression, ameliorating IR and normalizing blood glucose levels (30, 31). Parallel activation of AMPK by resveratrol further modulates FOXO1 and peroxisome proliferator-activated receptor (PPAR)-α/γ coactivator-1α (PGC-1α) (32, 33). Notably, AMPK may elevate NAD^+^ levels by stimulating its synthesis, subsequently activating SIRT1 and various downstream effectors (29). A recent correlative study revealed that resveratrol may upregulate SIRT1 expression, which subsequently activates liver kinase B1 (LKB1) - a pivotal threonine-protein kinase serving as the upstream regulator of AMPK. This molecular cascade facilitates AMPK phosphorylation through LKB1-mediated activation, thereby promoting the compound’s anti-hyperglycemic efficacy via enhanced cellular energy sensing and metabolic regulation (34). These findings further underscore the interplay between AMPK and SIRT1. Moreover, resveratrol has been shown to exerts dual activation of AMPK and SIRT1 through a phosphodiesterase (PDE)-inhibitory mechanism characterized by cyclic adenosine monophosphate (cAMP)accumulation, thus facilitating the synergistic signaling cascade (35).

Resveratrol’s glucoregulatory properties extend to the phosphatidylinositol-3-kinase/protein kinase B (PI3K/Akt) pathway, a central mediator of insulin signaling in IR (36). Under hyperglycemic conditions, insulin secreted by pancreatic β cells binding to its receptor triggers insulin receptor substrate (IRS) phosphorylation, initiating a signaling cascade through PI3K and Akt to regulate glucose uptake and metabolism (37). There was observed IR-associated impairments—elevated FBG and FINS levels, reduced IRS-1 and GLUT4 expression, and diminished PI3Kp110α and p-Akt levels in PCOS rats induced by dehydroepiandrosterone and a high-fat diet (38). Resveratrol exhibits pleiotropic regulatory effects on gene expression networks, including caspases, matrix metalloproteinases, adhesion molecules, and growth factors. It may also modulate the activity of several signaling pathways, including PI3K/Akt (39). Mechanistically, resveratrol enhances PI3K/Akt phosphorylation in renal tissues and downregulates mRNA and protein expression of phosphoenolpyruvate carboxykinase (PEPCK) and glucose-6-phosphatase (G6Pase), while PI3K inhibition abrogates p-Akt expression and reactivates FOXO1 via dephosphorylation (30). Interestingly, resveratrol exerts coordinated regulation of the PI3K/Akt pathway via SIRT1-dependent activation, which suppresses FOXO1 activity and expression by preventing its dephosphorylation, ultimately enhancing glucose uptake and insulin metabolism to maintain gluconeogenesis (40).

In conclusion, the therapeutic effects of resveratrol in ameliorating glucose metabolism abnormalities in PCOS through multi-pathway synergy rather than isolated mechanisms. Specifically, Its activation of either the PDE-cAMP-AMPK-SIRT1 axis and the PI3K-Akt signaling pathway highlights novel therapeutic strategies for addressing IR and glucose metabolism disturbances in PCOS.

### 2.2 Regulating the expression of factors involved in glucose metabolism

SIRT2, a member of the sirtuin family, plays a crucial role in metabolic homeostasis through post-translational modifications of target proteins, influencing gene expression and signal transduction pathways. Dysregulated SIRT2 activity is implicated in the pathogenesis of metabolic disorders, such as neurodegenerative diseases, tumors, diabetes, and cardiovascular diseases (41). Previous studies have shown that inhibiting SIRT2 may enhance the stability of glucokinase (GCK) regulatory proteins, accelerate aldolase A degradation, and inhibit β-cell insulin secretion via modulation of the Akt/glycogen synthase kinase-3β (GSK–3β)/β-catenin pathway, collectively reducing glycolytic flux (42). Conversely, SIRT2 activation may decrease acetylation of PEPCK 1, enhancing its stability, while deacetylating FOXO1 and PGC-1α to upregulate transcription of glucose metabolism-related bio-enzymes, resulting in the inhibition of gluconeogenesis (43). This dual regulatory capacity extends to hepatic glucose uptake, where SIRT2-mediated deacetylation of GCK regulatory proteins activates GCK, facilitating glucose phosphorylation (44). In high-fat diet and letrozole-induced PCOS rats model, Liang et al (45) found that resveratrol administration significantly decreased FBG, FINS, and HOMA-IR levels, concomitant with downregulated insulin-like growth factor-1 (IGF-1) and upregulated IGF-1 receptor expression. They also observed that resveratrol increased SIRT2 levels, upregulated the mRNA expression of glycolysis- enhancing enzymes—hexokinase 2 (HK2), lactic dehydrogenase A (LDHA), and pyruvate kinase M2 (PKM2)—and shifted the concentration of glycolytic products (reduced pyruvate and elevated lactate), promoting glycolysis (45).

Resveratrol demonstrates therapeutic potential in T2D by modulating microRNA (miRNA) expression networks. Mahjabeen et al (22) identified that six-month resveratrol supplementation significantly downregulates miR-21, miR-34a, and miR-375 in patients. Mechanistically, miR-21 suppression mitigates the production of hydrogen peroxide-induced reactive oxygen species (ROS) and regulates glycolytic flux in pancreatic stellate cells, contributing to its hypoglycemic effects (46). In parallel, miR-34a downregulation amplifies SIRT1 activation, restoring insulin sensitivity and glucose homeostasis (47). Furthermore, resveratrol may control glucose metabolism via downregulating miRNA-375, activating the cAMP/protein kinase A (PKA) signaling pathway, and reducing apoptosis of pancreatic β-cells, thereby maintaining adequate insulin secretion *in vivo (* 48). Notably, resveratrol also up-regulates the expression of miRNA-126 and miRNA-132, which synergistically improve glucose metabolism (22). Elevated miR-126 expression not only alleviates pancreatic β-cell damage and potentiates insulin secretion via PI3K/Akt signaling axis (49), also exhibits molecular interplay with the downregulation of high mobility group box 1 (HMGB1)—a critical inflammatory regulator, orchestrating oxidative stress and inflammation in the body (50, 51). Simultaneously, upregulation of miRNA-132 exerts potent suppressive effects on the activation of canonical inflammatory signaling cascades associated with IR, including nuclear factor kappa-B (NF-κB), tumor necrosis factor-alpha (TNF-α), NOD-like receptor (NLR), and Toll-like receptor (TLR) pathways (52).

Collectively, resveratrol counteracts IR-driven glucose metabolism dysregulation through dual mechanisms: epigenetic modulation of miRNA networks and SIRT2-dependent metabolic reprogramming. These findings underscore its multi-target capacity to restore glycemic control in PCOS.

## 3 Resveratrol improves abnormal lipid metabolism

Obesity, particularly central adiposity, is a prevalent comorbidity in PCOS, affecting 30%–70% of patients (53). Obese women with PCOS present more severe clinical phenotypes than their non-obese counterparts, including exacerbated menstrual irregularities, impaired glucose tolerance, T2D, and metabolic syndrome (53). Notably, even a 5%–10% reduction in modest weight achieves significant improvements in reproductive, metabolic, and psychological outcomes associated with PCOS (8). Consequently, the pivotal role of obesity in both the onset and perpetuation of PCOS cannot be overstated, as it profoundly impacts the clinical and endocrine manifestations of the condition (54). In individuals with PCOS, obesity serves a dual function. Firstly, elevated free fatty acid (FFA) levels impair glucose uptake in skeletal muscle and adipose tissue, further compromising insulin sensitivity and intensifying IR (55, 56). Secondly, excessive adipose tissue directly stimulates the ovaries and adrenal glands, destabilizing the hypothalamus-pituitary-ovarian (HPO) endocrine axis homeostasis. This instability results in HA, subsequently disrupting normal folliculogenesis and ovulation (56).

Dyslipidemia—characterized by elevated low-density lipoprotein (LDL), triglycerides (TG), and total cholesterol (TC), alongside reduced high-density lipoprotein cholesterol (HDL-C)—is prevalent in PCOS, with severity correlating positively with body mass index (BMI) (57, 58). Resveratrol, a polyphenol demonstrated to modulate cholesterol metabolism in T2D (59), may similarly ameliorate lipid abnormalities in PCOS by targeting pathways central to IR and adipocyte dysfunction.

### 3.1 Regulation of lipid metabolism-related signaling pathways

Adipocyte differentiation involves a transcriptional cascade orchestrated by CCAAT/enhancer-binding protein α (C/EBPα), sterol regulatory element-binding protein 1c (SREBP-1c), and PPAR-γ, which collectively drive the morphological transition of a fibroblastic cell shape to a spherical one (60). Of these, PPAR-γ is recognized as the primary regulator of adipogenesis, while C/EBPβ acts as an upstream activator, inducing the expression of both PPAR-γ and C/EBPα (60). PPAR-γ, a ligand-activated nuclear receptor, modulates lipid metabolism by regulating TG synthesis, lipid uptake, and cholesterol efflux (61). A meta-analysis of clinical trials revealed that resveratrol significantly reduces waist circumference, TC, LDL-C, and HDL-C levels in patients with obesity and diabetes (62). In high-fat-diet fed mice for eight weeks, resveratrol counteracted diet-induced elevations in TC and LDL-C while upregulating PPAR-γ and adiponectin (APN), suggesting the relationship between resveratrol, PPAR-γ, and cholesterol metabolism (63). In 3T3-L1 preadipocytes, resveratrol dose-dependently suppressed lipid accumulation and differentiation by downregulating protein expression of C/EBPβ, C/EBPα, and fatty acid-binding protein 4 (FABP4) (64).

IGF-1 modulates adipocyte metabolism through insulin-mimetic mechanisms, suppressing lipolysis and influencing carbohydrate-lipid crosstalk. Concomitantly, SIRT1, a central regulator of hepatic lipid and glucose homeostasis, governs metabolic flux between lipogenesis and fatty acid oxidation. Resveratrol stimulates SIRT1, upregulating PPAR-γ and its downstream fatty acid oxidation genes, thereby enhancing hepatic lipogenesis and elevating circulating IGF-1 levels (65). By activating SIRT1, resveratrol promotes cholesterol efflux through upregulation of liver X receptor α (LXR-α) and ATP-binding cassette transporter A1 (ABCA1). This is accompanied by regulation of lipoprotein lipase (LPL), enhancing lipid uptake (66). Additionally, resveratrol reduces lipoprotein(a) [Lp(a)] levels, hepatic 3-hydroxy-3-methylglutaryl-coenzyme A (HMG-CoA) reductase activity, and cholesterol ester transport protein concentrations while increasing apolipoprotein A1 (ApoA1), effectively ameliorating dyslipidemia (66).

Generally, resveratrol suppresses adipogenesis through inhibition of C/EBPβ, downregulates C/EBPα and PPAR-γ expression—key drivers of fibroblast-to-adipocyte morphological transition—and enhances IGF-1 signaling pathway activation. These multifaceted effects mechanistically appear to be mediated by SIRT1.

### 3.2 Regulating the expression of factors involved in lipid metabolism

Adipokines, a class of circulating hormones secreted by adipose tissue, mediate crosstalk between fat depots and metabolic organs such as the brain, liver, muscle, and immune system. Dysregulation of adipokine athways related to leptin and Asignaling is strongly implicated in obesity, T2D, and lipid metabolism disorders. Among these, leptin and APN are pivotal regulators of energy homeostasis and lipid metabolism (67).

Hyperleptinemia, a hallmark of obesity, induces leptin resistance via diminished hypothalamic sensitivity, perpetuating metabolic disturbances such as IR and cardiovascular disease. A recent study indicates that hyperleptinemia may independently predict the prognosis of cardiovascular disease, irrespective of obesity (68). Upon binding to leptin receptors (ObRs), leptin activates several signaling pathways, including Janus kinase/signal transducers and activators of transcription (JAK/STAT) pathway, extracellular signal-regulated kinase 1/2 (ERK1/2) in the mitogen-activated protein kinase (MAPK) pathway, and IRS/PI3K/Akt pathway (69). Furthermore, leptin is also involved in the AMPK and cAMP-response element binding protein (CREB)-regulated transcription coactivator (CRTC) pathway (70).

In contrast to leptin, circulating APN concentrations are reduced in obese individuals, which is a phenomenon hypothesized to play a pivotal role in the pathogenesis of atherosclerosis and cardiovascular diseases associated with obesity and the metabolic syndrome (71). The APN exerts its biological functions through ligand-receptor interactions with two transmembrane receptors, APN Receptor 1 (AdipoR1) and AdipoR2. Mechanistically, the adaptor protein containing pleckstrin homology domain (APPL1) orchestrates intracellular signaling cascades via its C-terminal phosphotyrosine-binding (PTB) and coiled-coil (CC) domains, which form critical structural interfaces with the cytoplasmic domains of both adiponectin receptors, thereby mediating the downstream effects of AMPK activation (72). Functioning as a metabolic integrator, APPL1 extends its regulatory capacity beyond direct AMPK modulation to bidirectionally govern PPAR-α expression—either through autonomous transcriptional control or AMPK-mediated crosstalk (73, 74). This dual regulatory axis coordinately activates mitochondrial β-oxidation machinery through transcriptional reprogramming, while enzymatically suppressing acetyl-CoA carboxylase (ACC) activity, thereby orchestrating a metabolic shift toward enhanced fatty acid catabolism and systemic lipid homeostasis (73, 74). Additionally, APN demonstrates pleiotropic effects on insulin signaling cascades. A study has revealed that APN potentiates insulin sensitivity through coordinated interactions with PI3K and Akt, establishing an APN-dependent insulin sensitization pathway (75). The lipoprotein-modulating capacity of APN manifests through dual hepatic mechanisms: Augmentation of HDL-C via transcriptional upregulation of hepatic ApoA1 synthesis, mediated through ABCA1-dependent which induces HDL assembly reverse cholesterol transport in peripheral tissues; Transcriptional activation of the PPAR-γ/LXR-α axis in macrophages, thereby enhancing HDL biogenesis (76). Especially, APN orchestrates TG homeostasis through complementary tissue-specific mechanisms: Suppression of hepatic apoC-III production upregulates LPL, enhancing TG catabolism; Upregulation of very low-density lipoprotein receptor (VLDLR) expression in skeletal muscle enhances TG-rich lipoprotein (VLDL) catabolism (77). Moreover, APN ameliorates IR may result in the reduction of VLDL production via inhibition of hormone-sensitive lipase (HSL) activity in adipose tissue, which blocks the breakdown of TG, decreasing FFA mobilization to the liver (77).

In patients with PCOS, there is a significant increase in serum leptin and a corresponding decrease in APN levels (78, 79). Emerging therapeutic research positions resveratrol as a dual modulator capable of normalizing this leptin-APN axis imbalance, suggesting its lipid-modulating efficacy in PCOS arises through pleiotropic mechanisms involving adipokines pathways related to leptin and APN (80).

Expanding beyond classical adipokines regulation, the therapeutic spectrum of resveratrol encompasses interactions with long non-coding RNAs (LncRNAs) a category of non-coding RNAs with a transcript length exceeding 200 nts, exhibiting tissue-specific and spatiotemporal-specific expression patterns. These regulatory RNAs serve as metabolic rheostats, critically governing glucose homeostasis, lipid turnover, and brown adipose tissue thermogenesis through epigenetic modulation (81). Recent studies have indicated that resveratrol may resveratrol exerts regulatory effects on lncRNAs expression profiles, notably upregulating MSTRG.7139 and MSTRG.9374 while downregulating MSTRG.12490 and MSTRG.13223 (82). More importantly, the anti-adipogenic properties of resveratrol may act on lncRNA, which in turn coordinated thrombospondin-4 (THBS4) and secreted frizzled-related protein 2 (SFRP2) activities, thereby simultaneously iregulating PI3K/Akt and Wnt pathway (82). Interestingly, *in vivo* and *in vitro* experiments further have demonstrated that resveratrol promotes fatty acid catabolism and suppresses lipid droplet formation and accumulation via enhancing the activity of AdipoQ-AdipoR1-AMPK-α and AdipoQ-AdipoR2-PPAR-α signaling pathways. triggering the upregulated expression of key lipolysis genes [adipose triglyceride lipase (ATGL), HSL] and fatty acid transfer genes [carnitine palmitoyl transferase 1 (CPT1), LPL], while downregulating the lipogenic gene ACC (82).

In conclusion, resveratrol potentially modulates lipid homeostasis through a tripartite mechanistic framework—suppressing the formation and accumulation of lipid droplets, and regulating cholesterol synthesis and excretion—which is mediated by leptin-APN endocrine axis while synchronizing lipid-metabolic LncRNAs.

## 4 Resveratrol improves hyperandrogenemia

HA, a hallmark of PCOS, manifests clinically as hirsutism, acne, alopecia, and ovulatory dysfunction (83). IR and subsequent hyperinsulinemia stimulate ovarian theca cells, enhancing steroid synthesis and promoting testosterone (T) secretion. Central obesity is associated with increased secretion of adipokines, which promote the release of pro-inflammatory mediators from peripheral tissues, elevating circulated androgen levels. Furthermore, insulin excess amplifies hypothalamic gonadotropin-releasing hormone (GnRH) pulsatility, elevating LH and further driving ovarian androgen production. Conversely, elevated androgens, in turn, act on pancreatic β-cells via androgen receptors, resulting in excessive insulin secretion. Moreover, androgens impairs subcutaneous adipose tissue to exacerbate IR by downregulating subcutaneous adipose HSL, reducing lipolysis, and promoting ectopic lipid deposition (84).

Accumulating clinical research confirmed resveratrol’s therapeutic potential in PCOS through coordinated endocrine modulation, notably reducing FINS and androgen levels (21, 85). Multi-tiered anti-androgenic effects of resveratrol may be exerted through divergent molecular pathways. Primarily, it suppresses ovarian theca cell proliferation via mevalonate pathway inhibition, achieved through transcriptional downregulation and enzymatic suppression of HMG-CoA reductase—the rate-limiting enzyme in cholesterol biosynthesis (21). Mechanistic divergence emerges in its regulation of cytochrome P450 family 17 subfamily A member 1 (CYP17A1), where resveratrol bypasses mevalonate modulation to directly impair the serine-threonine kinase/Akt cascade essential for steroidogenesis related protein activation, thereby impeding the conversion of progesterone to androgens (86).

Concurrently, resveratrol enhances APN secretion, which orchestrates dual metabolic-androgenic regulation: Potentiation of IRS-dependent PI3K/Akt signaling restores hepatic insulin sensitivity, attenuating compensatory hyperinsulinemia-driven theca cell hyperactivity (75). Moreover, resveratrol orchestrates multimodal physiological regulation through concerted modulation of core signaling networks, principally governing insulin signaling pathway dynamics, xenobiotic metabolism via cytochrome P450 enzyme systems, and steroidogenic axis coordination encompassing estrogen signaling, progesterone-mediated oocyte maturation, and steroid hormone biosynthesis, thereby systemically ameliorating HA and IR (87). Notably, resveratrol activates nesfatin-1-mediated lipolytic signaling and upregulates the expression of nesfatin-1, promotes lipolysis and glucose uptake, and reduces androgen secretion by improving obesity and IR (88).

Overall, resveratrol’s anti-androgen synthesis effect may be attributed to its direct interference with the expression of enzymatic sabotage of cholesterol-to-androgen conversion machinery (HMGCR, CYP17A1). Alternatively, it could indirectly decrease androgen production via modulating the HPO axis and recalibrating glucolipid metabolism through insulin sensitization and adipose tissue browning.

## 5 Resveratrol improves chronic inflammation and oxidative stress

PCOS is intrinsically associated with a chronic low-grade inflammatory milieu, wherein dysregulated proinflammatory cytokine networks perpetuate IR and adipocyte hypertrophy, thereby amplifying hyperinsulinemia-HA synergism. This pathophysiological triad is further compounded by oxidative stress [reactive oxygen species (ROS)-mediated endothelial and mitochondrial dysfunction], establishing a self-reinforcing cycle (89). A meta-analysis have recorded that resveratrol dismantle this vicious cycle (chronic inflammation and oxidative stress) in PCOS mice, and improve levels of androgen, gonadotropin, and vascular endothelial growth factor (VEGF), as well as reduce endoplasmic reticulum stress in PCOS patients (16). These findings suggest that a therapeutic convergence of resveratrol’s anti-inflammatory potency, antioxidant efficacy, and angio-modulatory properties positions as a pleiotropic agent capable of simultaneously targeting PCOS.

### 5.1 Anti-inflammatory and anti-oxidative stress

Previous research has founded that the serum levels of inflammatory markers, such as C-reactive protein (CRP), tumor necrosis factor α (TNF-α), interleukin 6 (IL-6), and IL-18, are significantly elevated in women with PCOS compared to healthy controls (89, 90). Similarly, these women also exhibit increased levels of oxidative stress indices such as malondialdehyde (MDA) and ROS alongside depleted antioxidant enzymes reserve, including glutathione S-transferase (GST), superoxide dismutase (SOD), catalase (CAT), and glutathione peroxidase (GSH-Px) (91). Clinical intervention trials reveal resveratrol’s capacity to recalibrate this redox-inflammatory axis: a 40-day regimen in PCOS patients (n=20) demonstrated significant reductions in IL-6, IL-1β, TNF-α, IL-18, NF-κB, and CRP (92).

Resveratrol orchestrates a multipronged anti-inflammatory and antioxidant strategy through precision modulation of redox-sensitive signaling hubs. Resveratrol attenuates NF-κB activation by blocking the phosphorylation and degradation of its inhibitory subunit, IκB, thereby preventing nuclear translocation of the NF-κB p65 subunit, which reduces the expression of inflammatory cytokines, including IL-1β, IL-6, and TNF-α (93). By inhibiting phosphorylation of p38 and ERK1/2 in the MAPK cascade, resveratrol further curtails inflammatory mediator production (93). Moreover, Resveratrol suppresses lipopolysaccharide (LPS)-induced overexpression of TLR4, dampening the TLR4/NF-κB/signal transducer and activator of transcription (STAT) signaling cascade and reducing synthesis of pro-inflammatory enzymes such as cyclooxygenase-2 (COX-2) (94).

In parallel, Resveratrol enhances the nuclear factor erythroid 2-related factor 2 (Nrf2) pathway, upregulating activating transcription factor 4 (ATF4) and ATF6, this suppresses endoplasmic reticulum (ER) stress markers, including CCAAT/enhancer binding protein homologous protein (CHOP), glucose-regulated protein 78 (GRP78), and X-box binding protein 1 (XBP1), while boosting the activity of antioxidant enzymes such as SOD, CAT, and GSH-Px, as well as controlling NAD phosphate (NADPH) oxidase-mediated ROS generation, reducing lipid peroxidation markers like MDA levels, and suppressing nitric oxide (NO) overproduction, thereby exerting anti-inflammatory and anti-oxidant effects (92, 95).

### 5.2 Reducing the expression of VEGF

Cyclical angiogenesis is critical for maintaining follicular growth, ovulation, and subsequent corpus luteum development and regression. Studies have found that primordial and primary follicles lack intrinsic vasculature, relying on the blood supply from interstitial blood vessels (96). As follicles begin to develop, microvasculature emerges, with its density continuously increasing until an extensive microvascular network is ultimately formed (96). VEGF, a highly pleiotropic growth factor, binds to high-affinity receptors on endothelial cells and plays a crucial role in controlling vascular permeability, sustaining the survival of newly formed blood vessels, and inducing certain organ-specific angiogenic factors (97). Secreted by ovarian granulosa cells, theca cells, and endometrium, VEGF is implicated in ovarian follicle formation and the normal reproductive process. Elevated serum VEGF concentrations induces abnormal ovarian angiogenesis, contributing to ovarian hyperstimulation syndrome, ovulatory disorders, and subfertility in women with PCOS, as well as gynecological diseases such as endometriosis (EMs) (98). Increased concentrations of VEGF in the ovary, serum, and follicular fluid in PCOS patients, particularly in obese individuals (99). This suggests that VEGF is involved in the pathogenesis of PCOS and indicates a potential association between VEGF and obesity.

Previous studies indicate that resveratrol exerts anti-angiogenic activity by transcriptional-epigenetic regulation of VEGF signaling networks (100). Clinical studies have shown that EMs patients treat with resveratrol for 12 weeks exhibited significant reductions in VEGF and TNF-α at both gene and protein levels, inhibiting ectopic endometrial invasion (101). Mechanistically, by suppressing NF-κB activation—via blocking IκB degradation and p65 nuclear translocation—resveratrol attenuates NF-κB-driven VEGF secretion and IL-8 production, mitigating inflammatory angiogenesis (102). In parallel, hypoxia-inducible factor-1 (HIF-1), a key transcriptional regulator of VEGF, is closely linked to ovarian androgen activity. Resveratrol may downregulate VEGF and HIF-1 gene expression in granulosa cells, disrupting the HIF-1/VEGF axis, alleviating HA-induced chronic inflammation and altering pro-angiogenic molecular pathways (85). This contributes to preserve ovarian reproductive potential by maintaining follicular microenvironment homeostasis.

In summary, resveratrol suppresses the expression of VEGF and is involved in both inflammatory responses and oxidative stress. Its anti-inflammatory and antioxidant properties may also contribute to alleviating symptoms associated with PCOS.

## 6 Resveratrol improves ovarian function

Ovulatory dysfunction, a primary characteristic of PCOS, is driven by interconnected pathological processes that disrupt follicular maturation and ovulation. Previous studies suggest that ovarian angiogenesis and granulosa cell autophagy play roles in the ovulation process (91, 103). Furthermore, oxidative stress, HA, and hormonal imbalance collectively impair the ovulatory function in PCOS (91, 103).

### 6.1 Regulation of reproductive-related hormones

The HPO axis is a highly coordinated neuroendocrine system integrating the hypothalamus, pituitary gland, and ovaries to regulate reproductive cyclicity. Central to this regulation is the pulsatile secretion of GnRH from the hypothalamus, which governs the release of FSH and LH from the pituitary gland. In a physiologically normal menstrual cycle, the pulsatile frequency of GnRH undergoes phase-dependent modulation: during the early follicular phase, GnRH pulses occur at intervals of approximately 90–100 minutes, favoring FSH-driven follicular recruitment and estrogen synthesis. As the cycle progresses to the late follicular phase, GnRH pulse frequency accelerates to 60-minute intervals, facilitating a shift toward LH dominance that culminates in the preovulatory LH surge, reflecting the transition in hormonal predominance from FSH to LH, as is required for successful ovulatory cycles (104).

In PCOS, however, diminished sensitivity of GnRH neurons to steroid hormone negative feedback results in increased GnRH pulse frequency, driving preferential pituitary LH hypersecretion and concomitant FSH suppression, resulting in an elevated LH/FSH ratio (105). The aberrant GnRH pulsatility and imbalanced LH/FSH ratio further disrupt ovarian follicular development, often stalling follicular maturation. Concurrently, high-amplitude and high-frequency LH pulses promote excessive androgen synthesis in theca cells, contributing to follicular atresia and the absence of dominant follicle selection. Elevated androgens further inhibit granulosa cell proliferation and suppress aromatase activity, reducing estrogen synthesis. This cascade blocks the transition of follicles from the preantral to antral stage, perpetuating the hallmark features of PCOS, such as oligo-ovulation and PCOM. A study demonstrates that resveratrol intervention in PCOS patients significantly reduces circulating LH and total T levels while restoring FSH concentrations, suggesting its capacity to rebalance HPO axis dynamics and ameliorate follicular stagnation (85, 106). These effects likely arise through resveratrol’s modulation of GnRH pulsatility and steroidogenic enzyme activity, highlighting its potential to disrupt the self-reinforcing hormonal imbalances central to PCOS pathophysiology.

Anti-Müllerian hormone (AMH) and FSH are pivotal biomarkers in PCOS, serving as indicators of oocyte competence and ovarian reserve (107). In PCOS, serum AMH levels are typically elevated due to the excessive accumulation of small antral follicles, reflecting both increased follicular recruitment and impaired selection of a dominant follicle. Within the ovary, AMH plays a critical role in folliculogenesis and the selection of the dominant follicle. Follicles exhibit increased sensitivity to FSH, and their further growth becomes dependent on gonadotropins during this selection process (108). AMH inhibits this FSH-dependent follicular growth. Consequently, elevated AMH levels reduce follicular sensitivity to FSH, leading to the accumulation of small antral follicles and contributing to anovulation in PCOS (108). Notably, Pratama G et al. (2) identified that AMH levels are positively correlated with the free androgen index and exhibit a direct positive association with the LH/FSH ratio, further highlighting the interplay of HA and gonadotropin dysregulation in perpetuating AMH-driven follicular arrest. Resveratrol demonstrates therapeutic potential in PCOS by rebalancing gonadotropin secretion and attenuating HA. Clinical studies report that resveratrol supplementation significantly reduces serum T, AMH, TNF-α, and MDA levels while elevating FSH concentrations in PCOS (85, 106, 109).Its potential mechanisms may involve correcting gonadotropin imbalances (e.g., normalizing the LH/FSH ratio), mitigating HA, alleviating chronic inflammation, and counteracting oxidative stress in ovarian follicles. By enhancing the integrity of granulosa cell layer and reducing educe prevalence of atresic follicles and follicular cysts, resveratrol promotes the transition of follicles from the preantral to antral stage, facilitating the development and maturation of dominant follicles (109). These effects are further reflected in the resolution of PCOM and restoration of menstrual cyclicity.

### 6.2 Regulation of granulosa cell proliferation and apoptosis

Resveratrol demonstrates multifaceted therapeutic potential in ameliorating PCOS by targeting granulosa cell (GC) dysfunction and mitochondrial anomalies. Bahramrezaie et al. (85) revealed that resveratrol suppresses VEGF and HIF-1 expression in GCs, enhancing the growth and development of high-quality oocytes, thereby increasing the rate of high-quality embryos. This aligns with findings by HUO et al. (110), where resveratrol restored glycolytic flux in PCOS rats by upregulating LDHA and PKM2, while augmenting SIRT1 activity. These effects correlated with normalized estrous cyclicity, increase the thickness of the GC layer, and reverse the decrease in GC proliferation and increase in cell apoptosis in the ovarian tissue of PCOS rats (110). Further mechanistic studies by DENG et al. (111) discovered resveratrol’s role in activating the SIRT1/AMPK pathway, which phosphorylates AMPK to inhibit intracellular autophagy via downregulation of Beclin-1 and upregulation of p62, thereby promoting GC growth and proliferation in PCOS rats. Additionally, it has been reported that resveratrol fosters the survival of GC in PCOS rats by activating the PI3K/Akt/mammalian target of rapamycin (mTOR) signaling pathway, which reduce the expression of intracellular autophagy-related proteins light chain 3I (LC3I), LC3II, and Beclin-1, suppressing the formation of autophagosomes (106, 112).

Mitochondria play a pivotal role in energy production, and mitochondrial dysfunction at the cellular level disrupts systemic metabolic homeostasis. In PCOS, ovarian tissues and GC is characterized by diminished expression of biogenesis regulators—PGC-1α, nuclear respiratory factor 1 (NRF-1) and mitochondrial transcription factor A (TFAM) —alongside increased mitochondrial DNA (mtDNA) damage and fragmentation, and ultrastructural abnormalities such as disorganized cristae, swelling, and vacuolization (113). These defects are coincided with decreased ATP production, diminished respiratory chain complex activity, and reduced mitochondrial membrane potential (MMP). Concurrently, oxidative stress markers (ROS and MDA) are elevated, mitochondrial dynamics are imbalanced (impaired fusion and excessive fission), and antioxidant capacity declines, exacerbating oxidative stress (114). Hu et al. (115) demonstrated that resveratrol concentration-dependently rescues cell integrity in PCOS GCs, like by improving mitochondrial morphology and function, increasing mitochondrial quantity and quality, and boosting intracellular ATP generation. Moreover, with mitochondrial functional recovery, resveratrol attenuates oxidative stress and apoptosis induced by mitochondrial damage, as evidenced by attenuated mitochondrial depolarization, restored MMP, and markedly reduced enzymatic activity and protein expression of caspase-3 and caspase-9. Collectively, resveratrol counteracts PCOS-associated GC dysfunction through coordinated modulation of angiogenic, metabolic, autophagic, and mitochondrial pathways.

### 6.3 Alleviating transzonal projection damage

Transzonal projection (TZP), specialized cytoplasmic extensions connecting granulosa cells with the oocyte, emerge as critical mediators of bidirectional communication during early folliculogenesis, becoming detectable coincident with zona pellucida formation. The number of TZP increases as oocyte growth progresses but rapidly diminishes at the time of ovulation (116). If TZP is compromised, the quality of the oocyte deteriorates, preventing its maturation (116). Chen et al. (117) observed that resveratrol restores TZP formation and function in PCOS models by enhancing calcium ion (Ca²^+^)/calmodulin (CaM) transmembrane transport to the cytoplasm. This activates calmodulin-dependent protein kinase IIβ (CaMKIIβ) phosphorylation, which stimulates actin monomer release and cytoskeletal remodeling, thereby re-establishing oocyte-granulosa communication and resolving ovulation defects.

In general, the ameliorative effects of resveratrol on ovulatory dysfunction in PCOS may be attributed to its ability to regulate hormone secretion within the HPO axis, modulate granulosa cell proliferation and apoptosis to promote follicular development, and enhance the generation of TZP (tight junction proteins) to mitigate TZP impairment and restore ovarian function.

## 7 Resveratrol regulates gut microbiota

The gut microbiota, a complex ecosystem of commensal microorganisms, plays a pivotal role in modulating host metabolic homeostasis, hormonal regulation, and immune-inflammatory responses. Emerging data implicates gut dysbiosis—a disruption in microbial composition and function—in the pathogenesis of diverse diseases, including reproductive and gynecological disorders (118). In PCOS, dysbiosis compromises intestinal barrier integrity by disrupting tight junction proteins, leading to increased intestinal permeability and systemic translocation of bacterial endotoxins. This endotoxemia triggers chronic low-grade inflammation, elevating pro-inflammatory cytokines, which may disrupt insulin signaling and fatty acid metabolism, further exacerbating IR and lipid metabolism irregularities.

A meta-analysis revealed that the most prevalent bacterial alterations in PCOS, including *Bacteroidaceae*, *Coprococcus*, *Bacteroides*, *Prevotella*, *Lactobacillus*, *Parabacteroides*, *Escherichia/Shigella*, and *Faecalibacterium prausnitzii* (119). Additional literature has discovered a depletion of *Lachnospira* and *Prevotella*, alongside an enrichment of *Bacteroides*, *Parabacteroides*, *Lactobacillus*, *Fusobacterium* and *Escherichia/Shigella* in women with PCOS (120). Likewise, an operational taxonomic unit (OTU3) within *Bacteroides*—sharing 99.2% sequence similarity with *Bacteroides acidifaciens*, a species inversely correlated with obesity—was markedly diminished in PCOS-like mouse models (121). These findings collectively elucidate that gut dysbiosis, marked by a decline in anti-inflammatory and short-chain fatty acid (SCFA)-producing bacteria, exacerbates metabolic and hormonal disturbances in PCOS. Resveratrol demonstrates prebiotic-like properties that rectify these microbial imbalances. Preclinical studies report that resveratrol increases *Bacteroidetes* and *Actinobacteria* while reducing *Firmicutes* at the phylum level (122), counteracting the Firmicutes/Bacteroidetes ratio elevation linked to obesity and IR. It also elevates *Bifidobacteriaceae* and suppresses *Streptococcus* at the family level (122). At the genus level, resveratrol enriches *Lactobacillus* and inhibits *Enterobacter* (122). Resveratrol suppresses the expression of lipogenic genes, including *Gpat1* (glycerol-3-phosphate acyltransferase 1), *Mogat* (monoacylglycerol O-acyltransferase), and *Pparg* (PPAR-γ), through microbiota-dependent mechanisms. This inhibition normalizes hepatic and adipose lipid accumulation, restoring systemic lipid homeostasis (123). By modulating the relative abundance of *Ruminococcus* (a genus linked to fiber fermentation and SCFA production) and *Clostridium* (associated with bile acid metabolism), resveratrol enhances insulin signaling via IRS-1 phosphorylation while suppressing mTOR expression and hyperactivation. This dual action repairs insulin receptor sensitivity and ameliorates hyperglycemia (123). Resveratrol restores the integrity of the intestinal barrier by altering the composition of gut microbiota via upregulating the expression of tight junction proteins, zonula occludens-1 (ZO-1) and occluding. It also attenuates systemic inflammation caused by the enhancement of TLR4 and myeloid differentiation factor 88 (MyD88), suppressing the release of inflammatory factors such as TNF-α and IL-1.

Resveratrol demonstrates the capacity to restructure gut microbiota composition, which may in turn alleviate the abnormal glucose/lipid metabolism, chronic inflammation, and oxidative stress associated with gut dysbiosis in PCOS pathogenesis.

## 8 Conclusion

**Figure 1:**
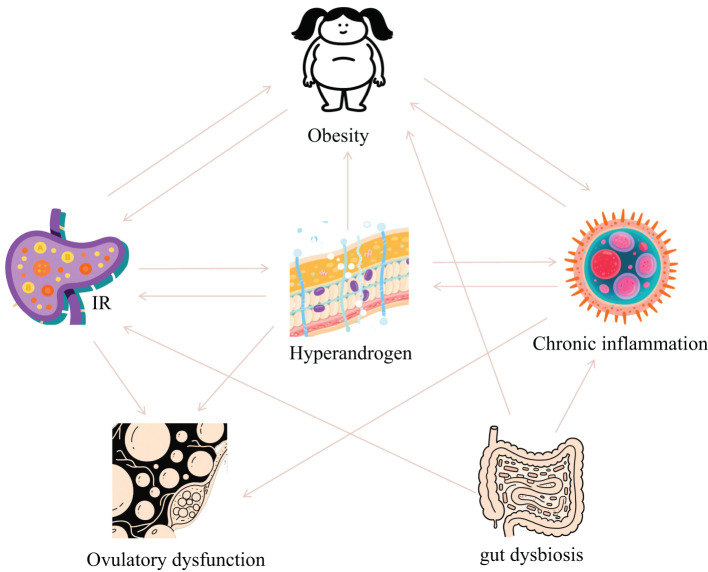
The relationship between pathological links of PCOS.

**Figure 2:**
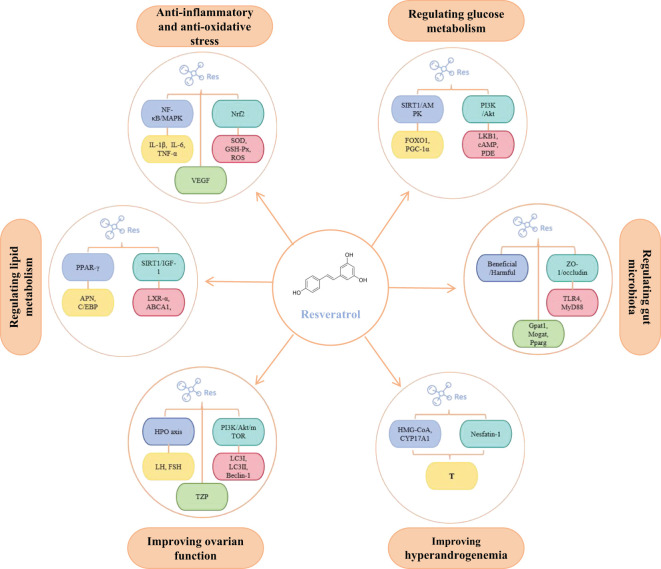
The main molecular targets of resveratrol in regulating PCOS. In addition, to ensure all figures meet the highest quality/resolution standards, we are uploading a PDF file containing the images.

PCOS is fundamentally characterized by interconnected pathological axes encompassing dysregulated glucose-lipid metabolism (manifesting as IR and obesity), HA, chronic inflammation with oxidative stress, and ovulatory dysfunction. These core pathophysiological components exhibit intricate bidirectional crosstalk, operating through mutually reinforcing mechanisms that collectively perpetuate the syndrome’s phenotypic heterogeneity and clinical progression (**Figure 1**). The accumulating preclinical and clinical evidence establishes resveratrol as a promising multi-target therapeutic agent for PCOS, offering potential advantages over conventional symptomatic management strategies. Its demonstrated efficacy in improving insulin sensitivity via reducing FINS by 31.8% and improving ISI by 66.3% in clinical trials, ameliorating dyslipidemia, reducing HA via decreasing testosterone and LH levels while increasing FSH, attenuating chronic inflammation and oxidative stress, restoring ovarian folliculogenesis and ovulation via normalizing estrous cycles and increasing high-quality oocyte rates, and modulating gut microbiota dysbiosis. Collectively, these actions target interconnected reproductive, metabolic, and inflammatory pathologies inherent to PCOS (**Figure 2**, **Table 1**). Crucially, resveratrol achieves these outcomes with a favorable safety profile, potentially circumventing the gastrointestinal intolerance associated with metformin, relapse risk after COCP discontinuation, and the teratogenicity concerns of anti-androgens. This pleiotropic activity underscores significant clinical utility, positioning resveratrol as a valuable adjunct or alternative therapeutic—particularly for patients intolerant to first-line agents or those seeking concurrent management of metabolic and reproductive sequelae.

**Table 1:** Regulation of resveratrol in PCOS.

Action		Pathways	Indicators	Reference
Regulating glucose metabolism	Relevant signals	SIRT1/AMPK	FOXO1↓, PGC-1α↓	(26–35)
			LKB1↑, cAMP↑, PDE↓	
		PI3K/Akt	p-PI3k↑, p-Akt↑, PEPCK↓, G6Pase↓	(36–40)
	Relevant molecules	SIRT2	HK2↑, LDHA↑, PKM2↑	(22, 41–52)
		miRNA	cAMP↑, PKA↑, HMGB1↓, NF-κB↓, NLR↓, TLR↓	
Regulating lipid metabolism	Relevant signals	PPAR-γ	APN↑, C/EBPβ↓, C/EBPα↓, FABP4↓	(60–66)
		SIRT1/IGF-1	LXR-α↑, ABCA1↑, LPL↓, Lp(a)↓, ApoA1↑	
	Relevant molecules	APN/leptin	APPL1↑, ACC↓, ABCA1↑, HDL-C↑,	(67–82)
			apoC-III↓, LPL↑, VLDLR↑, VLDL↓, TG↓	
		LncRNA	ATGL↑, HSL↑, CPT1↑, LPL↑, ACC↓	
Improving hyperandrogenemia	——	——	HMG-CoA↓, CYP17A1↓, Nesfatin-1↑, T↓	(83–88)
Improving chronic inflammation and oxidative stress	Anti-inflammatory and anti-oxidative stress	NF-κB/MAPK	p38↓, ERK1/2↓, IL-1β↓, IL-6↓, TNF-α↓	(89–95)
		TLR4/NF-κB/STAT	COX-2↓	
		Nrf2	ATF4↑, ATF6↑, SOD↑, CAT↑, GSH-Px↑ CHOP↓, GRP78↓, XBP1↓, MDA↓, ROS↓, NO↓	
	Reducing VEGF expression	NF-κB	VEGF↓, IL-8↓	(85, 100–102)
		HIF-1/VEGF	VEGF↓, HIF-1↓	
Improving ovarian function	Regulating HPO axis	——	LH↓, T↓, FSH↑, LH/FSH↓, AMH↓	(104–109)
	Regulating GC proliferation and apoptosis	VEGF/HIF-1	oocytes↑, embryos↑	(85, 106, 110–115)
		SIRT1	LDHA↑, PKM2↑	
		SIRT1/AMPK	Beclin-1↓, p62↑	
		PI3K/Akt/mTOR	LC3I↓, LC3II↓, Beclin-1↓	
		Mitochondria	NRF-1↑, TFAM↑, ATP↑, MMP↑, caspase-3↓, caspase-9↓	
	Alleviating TZP damage	Ca²^+^/CaM	CaMKIIβ↑, TZP↑	(116)
Regulating gut microbiota	——	Beneficial Bacteria/Harmful Bacteria	Bacteroidetes↑, Actinobacteria↑, Firmicutes↓ Bifidobacteriaceae↑, Streptococcus↓ Lactobacillus↑, Enterobacter↓	(118–123)
		Gut microbiota related mediators	Gpat1↓, Mogat↓, Pparg↓	
			IRS-1↑, mTOR↓	
		ZO-1/occludin	TLR4↓, MyD88↓, TNF-α↓, IL-1↓	

“↑”, Upregulation; “↓”, Downregulation.

Furthermore, future research should prioritize large-scale, long-term randomized controlled trials to definitively establish optimal dosing regimens, confirm sustained efficacy across diverse PCOS phenotypes—especially considering inherent heterogeneity and ethnic variations, and rigorously evaluate hard clinical endpoints such as live birth rates, prevention of T2D/cardiovascular events, and endometrial cancer risk reduction. Mechanistic studies should further delineate the relative contributions and potential synergies between its primary pathways—particularly SIRT1/AMPK, PI3K/Akt, and PPAR signaling—and evaluate its impact on specific patient subgroups stratified by biomarkers like AMH or gut microbial signatures. Concurrently, investigating the potential of resveratrol in combinatorial therapies, particularly with metformin where preliminary synergy exists, and exploring its role in adolescent PCOS management during pubertal transition represents critical translational avenues. Bridging the gap between robust mechanistic insights and definitive clinical validation through such integrated approaches is paramount to fully actualize resveratrol’s therapeutic potential and advance personalized clinical management for women with PCOS.
